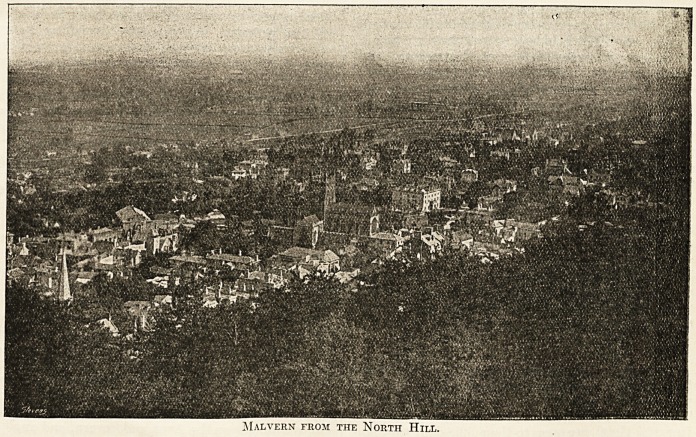# Home and Foreign Spas
*Previous articles in this series appeared in The Hospital of January 28, February 25, March 25, April 22, May 20, and June 3.


**Published:** 1911-06-17

**Authors:** 


### HOME AND FOREIGN SPAS.*

### VII. MALVERN.

Picturesquely situated on the sunny slopes of the Malvern Hills at an altitude of five hundred feet above the sea level, Malvern possesses all those characteristics so essential to a popular health-resort. The town, which fifty or sixty years ago was a mere cluster of hamlets, is to-day exceptionally attractive and well laid out with fine broad streets and side-walks sheltered by sweet-scented lime-trees. The greatest care is taken to keep the thoroughfares clean and clear of waste material, whilst the sanitary arrangements are on the most approved modern principles. Statistics show it to be one of the healthiest towns in the kingdom, singularly free from infectious diseases, with an average death-rate among residents as low as 10.8 per 1,000 of the population. The surrounding country provides some of the grandest scenery to be found in the beautiful county of Worcester, and on a bright day it is claimed that a view of from fourteen to sixteen counties is to be obtained from the hills above the town.

Malvern from the North Hill.

Malvern is on the Great Western Railway's main line to Hereford and the West, it is 127 miles from London, and about eight miles south-west of the city of Worcester. There are several trains daily from Paddington, with through carriages, which

*Previous articles in this series appeared in The Hospital of January 28, February 25, March 25, April 22, May 20, and June 3.

accomplish the journey in just over two and a half hours. It is also easily accessible from the chief centres in the Midlands, there being frequent trains from Birmingham, Derby, and York, whilst a variety of routes is open to those travelling from the North.

The climate is peculiarly tonic and invigorating, and the air dry and bracing with comparative freedom from dust and fog. The temperature is equable and the rainfall, notwithstanding the close proximity of the hills, by no means excessive, whilst thunderstorms are exceedingly rare. In consequence of the exhilarating effects of the atmosphere, its exceptional dryness and absence of extremes of heat and cold, the district is eminently suitable for phthisis and other affections of the chest. Patients suffering from rheumatism, anæmia, dyspepsia, and nervous disorders are also greatly benefited by a sojourn near the Malvern Hills.

### The Springs.

The water of Malvern, which rises directly from the syenitic rocks of which the Malvern Hills consist, is world-renowned for its purity and softness. There is probably no purer water obtained from natural sources, or one containing less inorganic salts, in existence. The analysis by Dr. Sheridan Muspratt, Principal of the Liverpool School of Chemistry, shows that the total salines are less than 4 grains in the imperial gallon.

In 1906 the water was again carefully analysed by Dr. John Thresh, who reported that it is one of the softest and purest waters found in nature, practically free from organic matter and bacteriologically of the highest degree of purity, and the same now as when examined by Dr. Muspratt fifty years ago. The importance of pure water in the treatment of various diseases arising from disorders of the digestive system can scarcely be over-estimated, and the absence of lime in Malvern water renders it of distinct service in all cases of calcareous degeneration of the arteries. So famous has this natural water become in consequence of its exceptional purity that large quantities are bottled by Messrs. W. & J. Burrow under the name of "Alpha" Brand Malvern Water, and despatched to all parts of the world. Besides the water in its natural state, this firm also supply Sparkling Malvern, which is simply plain water slightly aerated, and is useful for patients who suffer from rheumatism or gout.

### The Baths.

Malvern possesses two bathing establishments, thoroughly equipped with the most modern appliances and controlled by fully qualified attendants. The Imperial Baths, belonging to and adjoining the Imperial Hotel, are open to the public daily throughout the year, and comprise Vichy, Droitwich brine, Nauheim, electric light, massage douche, sitz, needle, spray and fango mud baths, as well as a large and elaborate swimming-bath. In connection with the brine baths, it is worthy of mention that a specially constructed railway truck is employed to convey the brine direct to Droitwich; also that the old practice of mixing hot water with the brine has long since been discontinued: the brine itself is now heated by steam, which enables the temperature to be more easily regulated to the requirements of the patient, without in any way reducing its therapeutic value. There is an excellent installation of baths at Dr. Fergusson's hydropathic establishment, including, in addition to those above enumerated, $x$-ray, static, sinusoidal, high-frequency and other treatments, but these are principally reserved to residents at the hydro and are open to a limited number only of non-residents. The cost of treatment depends largely upon the nature of the disease and kind of baths prescribed, but the fees at both establishments, in comparison with those charged at other resorts, are quite moderate.

### Accommodation and Amusements.

There is a number of first-class hotels, the Imperial probably being the largest and most sumptuously appointed. A special feature of this establishment is its underground subway to the station, a great convenience to residents in unpleasant weather. There are also many small private and family hotels and boarding-houses, and for those desirous of making a long stay a large selection of private apartments in every part of the town, where comfortable accommodation and board can be obtained at charges varying from 30s. to 50s. per week, according to the position and size of rooms. Conveyances of every description may be hired in all the principal thoroughfares, including motor-cabs, carriages, and bath chairs, which, in consequence of the hilly nature of the country, are drawn by small ponies.

A succession of theatrical performances by some of the best London touring companies, balls, concerts, and lectures are provided throughout the year in the Assembly Rooms. The Malvern Hydro. and many of the leading hotels frequently arrange musical evenings, dancing and various progressive games for the entertainment of residents. There is an exceptionally fine public library containing lending department, reference library, and news-room, a gentlemen's club, and a comfortable town club open to visitors at a nominal subscription.

The Manor Park, some ten acres in extent, situated in the very centre of the town, affords ample provision for such outdoor games as lawn tennis, croquet, bowls, and archery. Players' tickets are issued for the season, month, fortnight, or week. In addition, there is an excellent golf course of eighteen holes adjoining Malvern Wells Station and a separate nine-hole course for ladies with its own clubhouse. The Croome, Worcestershire, and Ledbury Hounds meet in the neighbourhood; boating is available on the Avon and Severn, and angling in the Teme, Severn, Wye, Lugg, and Frome, all within short distances of the resort. The roads are excellent for walking or cycling, and in the summer months there are motor and charabanc tours to the many places of scenic and historic interest in which the district abounds, but at all seasons of the year Malvern presents many charms peculiar to itself, difficult to equal, and almost impossible to surpass.

## Figures and Tables

**Figure f1:**